# Lineages evolved under stronger sexual selection show superior ability to invade conspecific competitor populations

**DOI:** 10.1002/evl3.80

**Published:** 2018-08-16

**Authors:** Joanne L. Godwin, Lewis G. Spurgin, Łukasz Michalczyk, Oliver Y. Martin, Alyson J. Lumley, Tracey Chapman, Matthew J. G. Gage

**Affiliations:** ^1^ School of Biological Sciences University of East Anglia Norwich NR4 7TJ UK; ^2^ Department of Entomology Institute of Zoology Jagiellonian University 30–387 Kraków Poland; ^3^ ETH Zurich Institute of Integrative Biology D‐USYS 8092 Zürich Switzerland

**Keywords:** experimental evolution, genic capture, introgression, mutation load, population fitness, *Tribolium*

## Abstract

Despite limitations on offspring production, almost all multicellular species use sex to reproduce. Sex gives rise to sexual selection, a widespread force operating through competition and choice within reproduction, however, it remains unclear whether sexual selection is beneficial for total lineage fitness, or if it acts as a constraint. Sexual selection could be a positive force because of selection on improved individual condition and purging of mutation load, summing into lineages with superior fitness. On the other hand, sexual selection could negate potential net fitness through the actions of sexual conflict, or because of tensions between investment in sexually selected and naturally selected traits. Here, we explore these ideas using a multigenerational invasion challenge to measure consequences of sexual selection for the overall net fitness of a lineage. After applying experimental evolution under strong versus weak regimes of sexual selection for 77 generations with the flour beetle *Tribolium castaneum*, we measured the overall ability of introductions from either regime to invade into conspecific competitor populations across eight generations. Results showed that populations from stronger sexual selection backgrounds had superior net fitness, invading more rapidly and completely than counterparts from weak sexual selection backgrounds. Despite comprising only 10% of each population at the start of the invasion experiment, colonizations from strong sexual selection histories eventually achieved near‐total introgression, almost completely eliminating the original competitor genotype. Population genetic simulations using the design and parameters of our experiment indicate that this invasion superiority could be explained if strong sexual selection had improved both juvenile and adult fitness, in both sexes. Using a combination of empirical and modeling approaches, our findings therefore reveal positive and wide‐reaching impacts of sexual selection for net population fitness when facing the broad challenge of invading competitor populations across multiple generations.

**Impact Summary**

Sexual selection, when competition and choice operate in the struggle to reproduce, could act either positively or negatively for sexually reproducing populations. If reproductive success in the face of strong sexual selection depends on being better at most things, then sexual selection could be positive in helping lineages to purge deleterious mutations and fix beneficial ones. By contrast, if reproductive competition leads to adaptations that cause conflict between males and females, or between sexual and nonsexual adaptations, sexual selection could create negative overall outcomes. To test these hypotheses, we experimentally evolved lines of flour beetles across 7 years of divergence in sexual selection. Strong sexual selection was applied by allowing competition between five males for polyandrous reproduction with a single female, and weak sexual selection was applied by enforcing reproduction from monogamous pairs. After 77 generations under these divergent regimes, we tested whether sexual selection had been a positive or negative force by trialing lines from either regime through a novel invasion challenge. Here, we introduced small groups of ‘colonists’ from polyandrous or monogamous backgrounds into larger competitor populations, and then tracked the spread of their genes. Individuals in the competitor populations all carried a visible genetic marker, which allowed us to measure the spread of any invasion through eight generations of the challenge. We found that individuals evolved through strong sexual selection had superior invasion abilities compared to the monogamous background, quickly spreading through and almost completely eliminating the marker population by the end of the experiment. Our results therefore showed that sexual selection was a generally positive force for lineage health and performance, improving ability across all life stages and in both sexes to enable invasion.

## Introduction

The evolutionary persistence of a population depends on relative fitness across a wide range of life history and reproductive traits, and differential success across all of these components of total fitness will determine whether lineages thrive or face extinction (Darwin 1859; Fisher 1930; Orr 2009). Sexual selection, when competition and choice create variance in reproductive success between individuals (especially males) (Darwin 1874; Andersson 1994), could have either positive or negative consequences for the net fitness of populations or lineages facing a broad range of ecological and evolutionary challenges. Genic capture theory (Rowe and Houle 1996) provides a mechanism through which sexual selection, by favoring enhanced individual condition, can improve the net fitness of a population or lineage. Genic capture proposes that reproductive success in the face of competition and choice ultimately depends on an individual's overall condition, which will depend on genome‐wide variation (Rowe and Houle 1996; Lorch et al. 2003; Tomkins et al. 2004; Whitlock and Agrawal 2009). Thus, if sexual selection ultimately operates widely across the genome, acting on the range of biochemical, physiological, morphological, and behavioral traits that combine and interact across a life history to dictate individual condition, it could be a positive force for net population and lineage fitness.

Individual condition will depend on the presence of both beneficial alleles across the genome, as well as the existence of deleterious alleles in the form of ‘mutation load’. All life is subject to the perpetual risk of mutation load: the accumulation of imperfect genetic variants that exist in all lineages, and which reduces net fitness in a population below that of one which is hypothetically load‐free and perfectly adapted to its environment (Haldane 1937; Muller 1950; Crow 1958; Simmons and Crow 1977; Agrawal and Whitlock 2012; Plough 2016; Simons and Sella 2016). Natural selection will remove deleterious mutations with detectable phenotypic effects, however, mutations of weak effect are much harder to eliminate, but can sum across multiple loci and individuals to create a significant total genetic load (Fisher 1930; Haldane 1937; Muller 1950; Kimura et al. 1963; Felsenstein 1974; Charlesworth et al. 1993; Lande 1994; Lynch et al. 1995; Crow 1997). Sexual selection and genic capture could be a crucial force for limiting this mutation load, if reproduction in the face of intraspecific competition and choice is achieved by more individuals within a population that carry relatively less genetic load (Manning 1984; Whitlock 2000; Lorch et al. 2003; Whitlock and Agrawal 2009). This reasoning led Agrawal (2001) and Siller (2001) to theorize that longer‐term benefits arising from the purging of mutation load via sexual competition and mate choice could counter the significant costs of sex.

Despite these theorized benefits, opposing logic proposes that sexual selection creates conflicts leading to negative impacts on individual, and therefore population and lineage, fitness. Depending on how it acts within both sexes, sexual selection might create conflicting tradeoffs with naturally selected optima, biasing investment toward traits that benefit individual success within competitions for reproduction, at the expense of investment in traits such as those improving adult survival or offspring viability (Lande 1980; Kirkpatrick and Ryan 1991; Houle and Kondrashov 2001). Sexual selection might also favor the evolution of traits enabling individual reproductive success in one sex at the expense of that in the other, for example, in conflicts where competition between males reduces the reproductive fitness of the female, which they are competing over (Parker 1979; Arnqvist and Rowe 2005). Sexual conflict might also operate within the genome, when trait optima for one sex do not match those of the other, generating intralocus conflict that then constrains the potential fitness of individuals and populations (Parker 1979; Arnqvist and Rowe 2005; Bonduriansky and Chenowith 2009; Doorn and Sander 2009; Arnqvist and Tuda 2010). There are therefore a number of possible routes for sexual selection to create conflicts and tradeoffs, which burden the potential fitness of individuals, summing to a reduction in net fitness of the whole population (henceforth ‘population fitness’).

**Figure 1:**
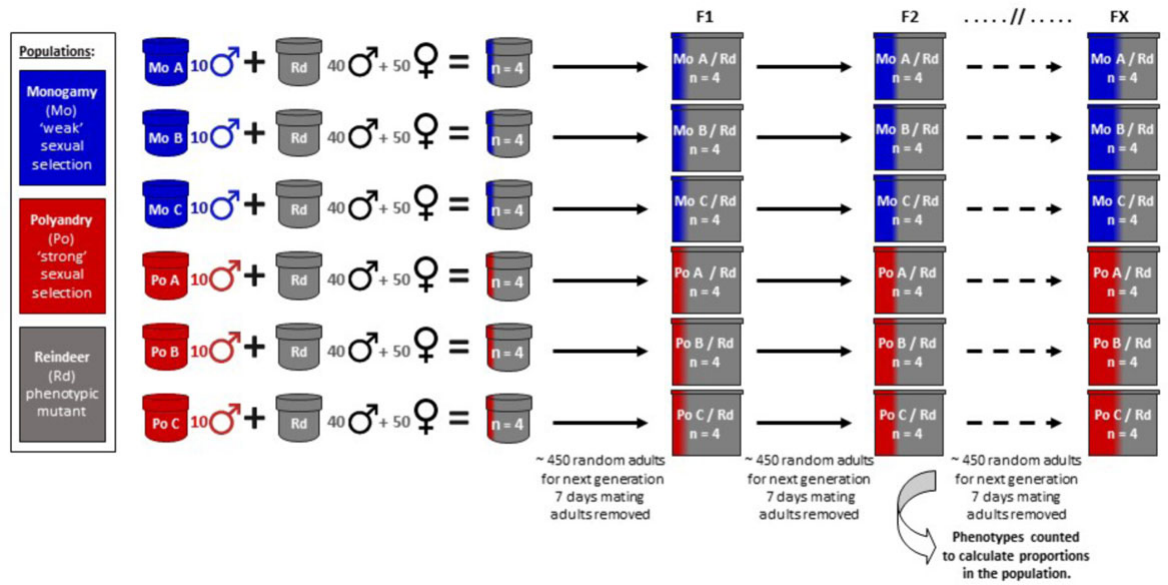
**Design of the invasion and introgression assay**. Ten males from contrasting Monogamous and Polyandrous sexual selection regimes (previously under experimental evolution for 77 generations) with a wild type phenotype, were initially introduced into competitor populations containing 90 adults of the Reindeer phenotypic mutant. The proportion of the population expressing the wild type phenotype was used in each adult generation as a measure of the extent of introgression (*n* = 4 replicates × 3 independent lines per sexual selection regime = 24 populations in total, counting and scoring almost 80,000 adults).

Here, we employ experimental evolution followed by a multi‐generational invasion experiment to test between these competing ideas using the model insect *Tribolium castaneum*, and determine whether sexual selection has positive or negative influences on net lineage fitness. We compare lineage net fitness through a broad ecological and evolutionary challenge that measured the ability of populations evolved under divergent strengths of sexual selection to colonize, outcompete, invade, and then introgress conspecific competitor populations (Fig. 1). This competitive invasion challenge, across multiple individuals, traits, life‐stages, and generations, provides a holistic test of overall population fitness; to invade successfully across multiple generations, adults of both sexes and their offspring must be, on average, competitively superior across a diverse range of traits affecting the whole life history (Agrawal and Whitlock 2012; Barrett et al. 2016). If, on the other hand, sexual selection focuses on a subset of traits and phenotypes that bias investment into reproductive competition at the expense of wider performance, and/or there is sexual conflict between male and female optima (Parker 1979; Arnqvist and Rowe 2005), we might expect stronger sexual selection to create lineages and populations that show reduced net fitness when faced with the diverse challenges of multi‐generational invasion and introgression.

Having empirically measured the rates and extents of invasion by conspecific colonists from different sexual selection backgrounds, we then explore the possible traits that enable successful introgression across multiple generations. First, we conduct simple fecundity tests in benign, noncompetitive conditions, in order to test whether variation in mating, fertility, or fecundity in either sex has evolved under different strengths of sexual selection, and also to determine whether any barriers to hybrid introgression exist between the invading and competitor *T. castaneum* strains. Second, we use population genetic simulations to model the progress of introgression across the eight generations of our experiment in order to reveal the likely drivers. Because of the specific design of our invasion experiment and knowledge of our genetic marker's inheritance, we were able to differentiate between three key fitness drivers explaining our results, depending on contributions generated by variance in: (1) adult male reproductive success, (2) adult female reproductive success, and (3) offspring performance and viability. Our experiment showed that invasion was achieved more rapidly by populations from lineages that had evolved under stronger sexual selection, gaining almost complete introgression by the end of the multigenerational challenge. Follow‐up tests revealed no differences in male or female fertility or fecundity between weak and strong sexual selection backgrounds, and no barriers to hybrid introgression between strains, indicating that simple reproductive potential and fertility (in the absence of competition and selection) had not limited invasion by Monogamous regime backgrounds. Population genetic simulations indicated that the superior ability of our stronger sexual selection backgrounds to invade could only be explained if sexual selection had improved (1) offspring performance across the juvenile stages, and (2) reproductive success by adults of both sexes, and not just male competitiveness. Using a combination of experimental evolution, an invasion assay measuring net fitness in evolutionary and ecological contexts, and subsequent simulations, our study therefore reveals that sexual selection improves net population fitness when facing the broad challenge of invading competitor populations across multiple generations.

## Methods

### BEETLE STOCKS AND CULTURE

*T. castaneum* were of the commonly‐used Georgia 1 (GA1) wild type (*WT*) strain (Haliscak and Beeman 1983), and a standard dominant phenotypic marker strain, Reindeer (*Rd*) (Dawson 1984; Lewis et al. 2005; Tregenza et al. 2009), which have distinctive swollen antennae. Both stocks originated from the Beeman Lab (United States Department of Agriculture). All beetles were maintained throughout under standard conditions of 30°C and 60% humidity, with a food medium consisting of 90% organic white flour, 10% brewer's yeast, and a layer of oats.

### EXPERIMENTAL EVOLUTION UNDER STRONG VERSUS WEAK SEXUAL SELECTION

Sexual selection regimes varied the mating system applied to the adult life stage. An enforced Monogamy (one male to one female) regime was contrasted with a Polyandrous regime (five males to one female) which applied opportunities for sexual selection (for full details see (Demont et al. 2014; Lumley et al. 2015). Within‐line replication of Monogamous pairs, 20 pairs per line, and Polyandrous groups, 12 groups per line, equalized the theoretical effective population size (Wright 1931) between regimes (*N_e_* = 40). Genetic testing using multiple microsatellites confirmed that neither regime had experienced differential genetic bottlenecking, with equal levels of heterozygosity in Monogamous and Polyandrous lines (Lumley et al. 2015). Three independent lines within either regime were maintained. For each line, at every generation, individuals sexed as pupae were used to then create adult pairs/groups in fresh medium for 7 days of mating, fertilization and oviposition. Monogamous pairs were maintained and mated in containers containing 2 g flour medium, and Polyandrous groups were maintained and mated in containers with 6 g flour medium We therefore allocated 1 g flour medium per adult in both regimes, with the breeding conditions in both being different to those in the subsequent invasion challenge (see next section). After reproduction, adults were removed and eggs/larvae pooled from the multiple pairs or groups within each line, with the flour medium increased and equalized between regimes to 100 g, and left to develop under standardized conditions until pupae were ready for the next generation.

### INVASION AND INTROGRESSION ASSAY

Invasion and introgression were assayed after 77 generations of experimental evolution. Groups from either Monogamous or Polyandrous backgrounds were introduced into Reindeer marker (*Rd*) competitor populations, and their spread tracked across eight generations (Fig. 1). The *Rd* mutation is dominant and maintained homozygous within the *Rd* strain, therefore, any offspring sired by introduced males from *WT* sexual selection backgrounds were not visible in the F1 generation. For the *WT* phenotype to be expressed, individuals must be homozygous for the *WT* allele. Comparison of the extent of invasion therefore began in the F2 generation, once both male and female offspring from the initial sexual selection *WT* introduction had potentially achieved reproduction. *Rd* individuals had a standardized sexual selection background consistent with natural populations that are promiscuous (Sokoloff 1972; Fedina and Lewis 2008; Michalczyk et al. 2011; Lumley et al. 2015).

At the start of the invasion assay, all adults were 10–14 days post‐eclosion and unmated. Competitor populations were created consisting of 50 female and 40 male *Rd* adults, each of which we colonized with 10 sexual selection *WT* background males. All populations therefore started with 100 adults in a 1:1 sex ratio. Four replicate populations (1 to 4) were created within each of the three independent sexual selection lines (A, B, and C) for both regimes (Monogamy versus Polyandry), so the experiment was run across *n* = 24 populations (4 replicate populations × 3 independent lines = 12 populations × 2 sexual selection regimes). Within the Parental generation for each population, adults were placed in 100 g fresh medium (1 g per adult) for 7 days mating and oviposition, after which they were removed and eggs/larvae were left under standardized conditions for 32–35 days allowing all the offspring to develop into adults. For each subsequent generation, all adults were sieved out of the population, mixed in a petri dish, and then a random subsample of ∼450 adults (range = 167–586, mean = 461) taken from each with an eppendorf tube and placed in 250 g fresh medium for 7 days mating and oviposition. It was not possible to distinguish between antennal phenotypes at this subsampling stage without microscopy, and the adult beetles were completely mixed, preventing any bias for or against *WT* or *Rd* phenotypes. After 7 days, these adults were then removed and frozen for counting and scoring (a total of almost 80,000 adults across the assay), while eggs/larvae were left to develop. A 100 g of additional food was provided 14 days into larval development for all populations, ensuring consistent and finite food resource throughout the invasion assay. The frozen adults were then counted and identified between sexual selection regime background (*WT*) phenotypes and *Rd* phenotypes by examining antennal morphology under a dissecting microscope. The extent of population invasion at each generation was measured as the proportion of individuals in the population exhibiting the *WT* phenotype, which will be conservative because of the dominant status of the *Rd* phenotype (i.e., heterozygotes were scored as the *Rd* phenotype).

### FERTILITY AND FECUNDITY POTENTIAL

To assess whether any variation in invasion and introgression could be driven primarily by fertility or fecundity differences between Polyandrous and Monogamous backgrounds, relative to *Rd* strain adults, we measured reproductive output in non‐competitive breeding conditions from intra‐ and inter‐strain male × female crosses using individuals from Monogamous and Polyandrous regimes after 95 generations of experimental evolution, and the *Rd* stock population. As in the invasion challenge, offspring production following 7 days of mating and oviposition was used to measure fertility/reproductive output, but under noncompetitive pair‐breeding conditions so that reproductive potential could be assessed. All adults were 10–14 days post‐eclosion and unmated. Pairs were placed together in 2 g fresh medium for 7 days to mate and oviposit, then adults removed, and eggs/larvae developed in 5 g additional fresh medium into adult offspring for counting. We carried out trials for (a) intra‐strain *Rd* pairs (*n_Rd_*_total_ = 45), (b) intra‐lineage SS pairs (*n* = 9 pairs × 3 independent lines = 27 pairs in total per sexual selection regime (*n*_SS total_ = 54)), and (c) inter‐strain crosses between SS males and *Rd* females (*n* = 21–24 pairs per independent line per sexual selection regime (*n_Rd_*_x SS total_ = 139). To assay additional potential fertility or incompatibility impacts in the second generation of invasion, ‘hybrid’ offspring from these inter‐strain crosses were sexed and the reproductive fitness measured of (d) hybrid sons crossed with *Rd* females (*n* = 22–24 pairs per independent line per sexual selection regime (*n*_hybrid son total_ = 138)) and (e) hybrid daughters crossed with *Rd* males (*n* = 21–24 pairs per independent line per sexual selection regime (*n*_hybrid daughter total_ = 138)) (Fig. 3).

### STATISTICAL ANALYSES

All analyses were conducted in R ‐ 3.4.2 (R Core Team 2015), with ‘plyr’ (Wickham 2011), ‘pastecs’ (Grosjean et al. 2014), ‘car’ (Fox and Weisberg 2011), and ‘stats’ (R Core Team 2015) packages used for data exploration, descriptive statistics, and testing assumptions. Figures were created using ‘ggplot2’ (Wickham 2009) and ‘gridExtra’ (Auguie and Antonov 2017). Generalized Linear Mixed Models (GLMMs) were constructed using the ‘glmmADMB’ (Fournier et al. 2012; Skaug et al. 2016) and ‘R2admb’ packages (Bolker et al. 2015), with a negative binomial error distribution, which was necessary due to overdispersion in the data. Models were fitted by maximum likelihood, and likelihood ratio tests and AIC values were used to compare models with and without factors of interest (Crawley 2013). GlmmADMB has two options for fitting a negative binomial model which both incorporate an additional parameter into the mean, variance relationship to account for overdispersion; (i) nbinom, where variance = mean (1+ mean/dispersion parameter) or (ii) nbinom1, where variance = mean × scale parameter (Bolker 2010; Fournier et al. 2012). Each option was tried and the model with the lowest AIC was selected.

Invasion and introgression, measured as the proportion of wild‐type phenotype in replicate populations across generations, was compared between sexual selection backgrounds by constructing a GLMM with a negative binomial error distribution. A maximal model was fitted with treatment (Monogamy or Polyandry) and generation (2 to 8) entered as fixed effects. Generation was also entered as a random effect to account for temporal pseudoreplication, and population (1 to 4) was nested within independent line (A, B, or C) as a random effect, to account for the hierarchical design (Crawley 2013).

Fertility and fecundity, measured as offspring production from 7 days mating and oviposition, was compared between *Rd* and *WT* strains, and between Monogamous and Polyandrous sexual selection regime backgrounds using GLMMs with a negative binomial error structure. Strain (*Rd* versus *WT*), and/or sexual selection background (Monogamy versus Polyandry) were entered as fixed effects and replicate line (A, B, or C) as a random effect.

### POPULATION GENETIC SIMULATIONS

Population genetic simulations, representing a simplified model of the invasion and introgression assay, were created in R. We simulated starting populations of 50 *Rd* females, 40 *Rd* males and 10 *WT* males, as per the experiment. Each generation, males and females were paired, and offspring genotypes were simulated under Mendelian inheritance, with 52 offspring per pair, corresponding to the mean observed number of offspring per female in small groups (Godwin and Gage unpubl. data). Thirty percent of the resulting offspring were allowed to survive to adulthood, and 230 males and 230 females (corresponding to the mean number of adults used to initiate each generation in the experiment) were recruited into the subsequent breeding population.

Three components of fitness were varied between *Rd* and *WT* genotypes: (1) male reproductive success, (2) female reproductive success, and (3) offspring performance and viability. The key difference between male and female reproductive success in the model was that *WT* males were present in the first generation, whereas *WT* females and their reproductive impact could only arise in subsequent generations, which meant that the model was especially sensitive to differences between male and female contribution to net fitness at the earlier stages of each simulation. To simulate overall differences in fitness, we created the parameter α, which represents the relative fitness of *WT* compare to *Rd* genotypes (i.e., α = 2 is a twofold increase in fitness of *WT* relative to *Rd*). To simulate differences in male reproductive success, we randomly sampled males as a function of α prior to pairing. Sampling was with replacement, and was weighted with probabilities 1/(α + 1) and α/(α + 1) for the *Rd* and *WT* males, respectively. This same procedure was used to simulate differences in female reproductive success, but only coming into effect after the first generation. Differences in offspring survival were simulated by weighting the random sampling of surviving offspring, again using probabilities of 1/(α + 1) and α/(α + 1) for the *Rd* and *WT* males, respectively.

Each simulation was run for eight generations, as per the experiment, with 500 replicates. For each generation, we calculated the proportion of *WT* homozygote genotypes in the adult breeding population, and compared this against the observed proportion of *WT* phenotypes. Eight separate sets of simulations allowed different combinations of fitness components to increase in *WT* relative to *Rd* genotypes: (i) equal fitness, (ii) male reproductive success only, (iii) female reproductive success only, (iv) both male and female reproductive success, (v) offspring performance and viability only, (vi) male reproductive success and offspring viability, (vii) female reproductive success and offspring viability, and (viii) male and female reproductive success, and offspring viability. For each simulation (excluding the “equal fitness” treatment), we also simulated four levels of *WT* fitness relative to *Rd* (α = 1.5, α = 2, α = 5, and α = 10).

The simulations involved some simplifications. First, we considered fitness to be fully linked to the *Rd* genotype. In reality, fitness becomes decoupled from the *Rd* genotype as the experiment progresses and, as a result, for a given value of α, our simulations likely overestimate the rate of *WT* introgression. Second, in simulations where multiple components of fitness were allowed to vary between *Rd* and *WT* genotypes, we used the same value of α for all fitness components. Finally, we simulated heterozygotes as intermediate in fitness between *WT* and *Rd* homozygotes.

## Results

### INVASION AND INTROGRESSION ABILITY

**Figure 2:**
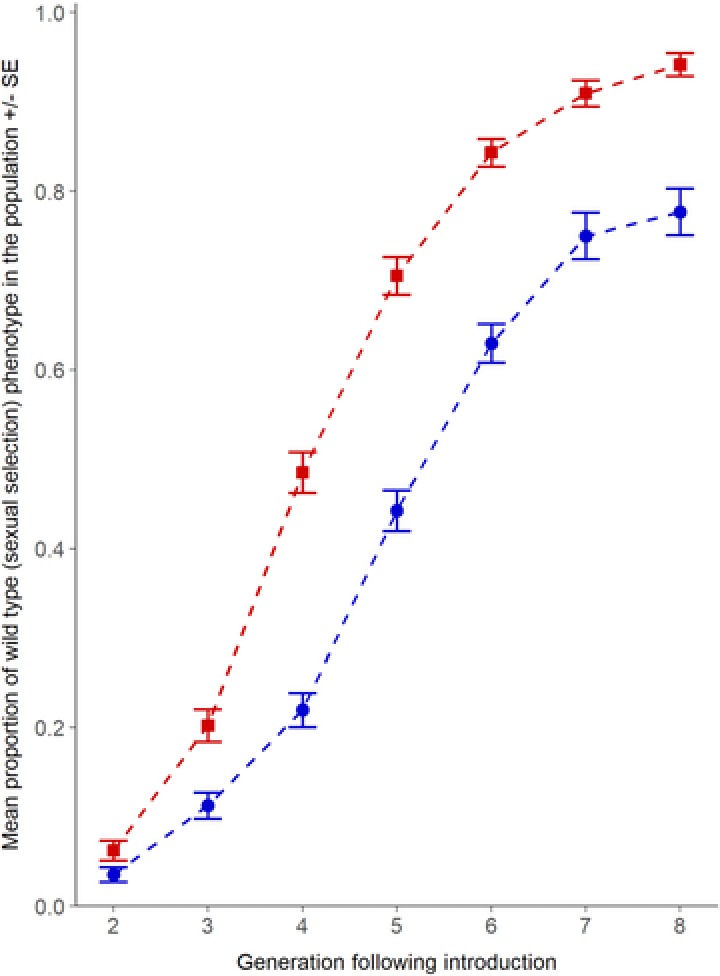
**Invasion into intraspecific competitor populations across eight generations by individuals sourced from contrasting histories of sexual selection (Monogamy (blue circles) vs. Polyandry (red squares))**. Data grouped by sexual selection regime (*n* = 4 replicate populations × 3 independent lines per regime). Extent of invasion was scored by levels of introgression of wild type alleles into Reindeer marker populations. The proportion of wild type phenotypes in experimental populations differs significantly between Polyandrous and Monogamous sexual selection regime backgrounds and across generations (negative binomial GLMM: χ^2^_(1)_ = 5.15, *P* = 0.02).

Following 77 generations of experimental evolution, we found that individuals introduced from evolutionary backgrounds exposed to stronger sexual selection under Polyandry (five males competing per female) invaded novel competitor populations across multiple generations more rapidly and completely, compared to individuals derived from histories of weak sexual selection under Monogamy (Fig. 2 and Table 1). As invasion proceeded across subsequent generations, indicating that the *WT* strain was generally superior to the *Rd*, the differences between Polyandrous and Monogamous backgrounds became more evident, reaching a maximum of 34% in generation 5 (Table 1). Thereafter, rate of invasion from the Polyandrous backgrounds decelerated as the *WT* allele moved closer to fixation above 90% at the *Rd* marker locus. By contrast, introductions from an evolutionary history of Monogamy exhibited a greater lag phase at the start of invasion, before exponential population growth commenced. Monogamous background invaders subsequently achieved a slower rate and level of introgression compared with colonizations from the stronger sexual selection background (Fig. 2). By generation eight, introgression from both backgrounds had plateaued (Fig. 2), with the *WT* allele carried at the *Rd* marker locus by 94% of the offspring in the populations invaded by the Polyandrous regimes, but in only 78% of individuals within populations invaded by the Monogamous regimes (Fig. 2).

**Table 1:** Summary of invasion rates into intraspecific competitor populations by individuals sourced from contrasting histories of sexual selection (SS)

	Mean (± SE) proportion of SS phenotype in the population		Outcome of maximal model (negative binomial GLMM)			
Generation	Monogamy	Polyandry		Estimate (SE)	*z*	*P*
**2**	0.04 (± 0.01)	0.06 (± 0.01)				
**3**	0.11 (± 0.01)	0.20 (± 0.02)				
**4**	0.22 (± 0.02)	0.49 (± 0.02)	**Intercept**	3.72 (0.15)	24.92	<0.001
**5**	0.44 (± 0.02)	0.79 (± 0.02)	**SS regime**	0.49 (0.15)	3.37	<0.001
**6**	0.63 (± 0.02)	0.84 (± 0.02)	**Generation**	0.24 (0.02)	13.74	<0.001
**7**	0.75 (± 0.03)	0.90 (± 0.01)				
**8**	0.78 (± 0.03)	0.94 (± 0.01)				

### FERTILITY AND FECUNDITY POTENTIAL

**Figure 3:**
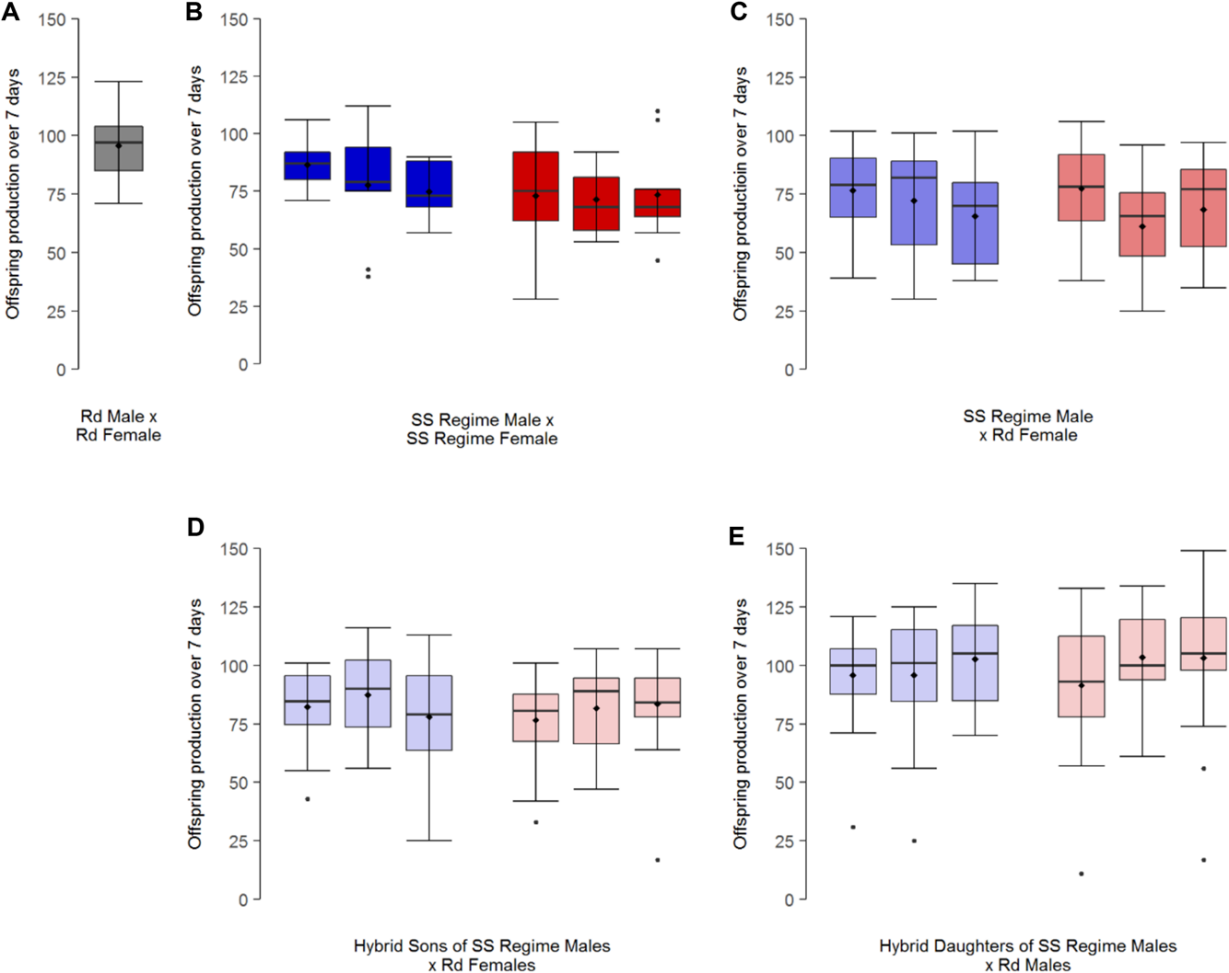
**Fertility and fecundity potential of intra‐ and inter‐strain breeding pairs from Reindeer (*Rd*) (grey), Monogamous (blue) and Polyandrous (red) sexual selection (SS) regime beetles, and hybrid sons and daughters thereof (decreasing color saturation represents dilution of sexual selection genetic background). (**A) *Rd* within‐strain pairs. (B) SS within‐strain pairs. (C) SS male and *Rd* female between‐strain pairs. (D) Hybrid SS × *Rd* sons paired with *Rd* females. (E) Hybrid SS × *Rd* daughters paired with *Rd* males. *Rd* reproductive output was ∼25% greater than SS, but there were no differences in fertility or reproductive fitness between Monogamous and Polyandrous backgrounds in either within‐ or between‐strain pairs. Data are grouped by independent line: (A) *n* = 45 pairs, (B) *n* = 9 pairs per independent line in either sexual selection regime (*n*_total_ = 54), (C–E) *n* = 21–24 pairs per independent line in either sexual selection regime. For all plots, horizontal lines indicate the median, boxes indicate the interquartile range (IQR), whiskers indicate points within 1.5 IQR, and any data not included in the box and whiskers are shown as outliers (small filled circles). A filled diamond indicates the mean.

Despite the evidence that *WT* strains invaded successfully, the fertility and reproductive fitness of noncompetitive breeding pairs from the *Rd* × *Rd* pairs was 25% higher (96 ± 2; mean ± SE) than that of *WT* strain pairs from either sexual selection regime (76 ± 3) (negative binomial GLMM: χ^2^_(1)_ = 10.34, *P* = 0.006). Within the *WT* strain, there were no differences in reproductive output or fertility between Monogamous and Polyandrous backgrounds, and no differences when males from either regime were crossed with *Rd* females (negative binomial GLMM: χ^2^_(1)_ = 0.34, *P* = 0.56). In addition, there were no differences between the sexual selection regimes when comparing reproductive output or fertility of *Rd* females paired with *WT* × *Rd* hybrid sons (negative binomial GLMM: χ^2^_(1)_ = 0.40, *P* = 0.53), or when comparing the reproduction of *Rd* males paired with *WT* × *Rd* hybrid daughters (negative binomial GLMM: χ^2^_(1)_ = 0.12, *P* = 0.73) (Fig. 3). Therefore, we find no evidence that variation in fertility or potential reproductive output of Polyandrous and Monogamous sexual selection histories could explain the differences in population invasion ability.

### POPULATION GENETIC SIMULATIONS

**Figure 4:**
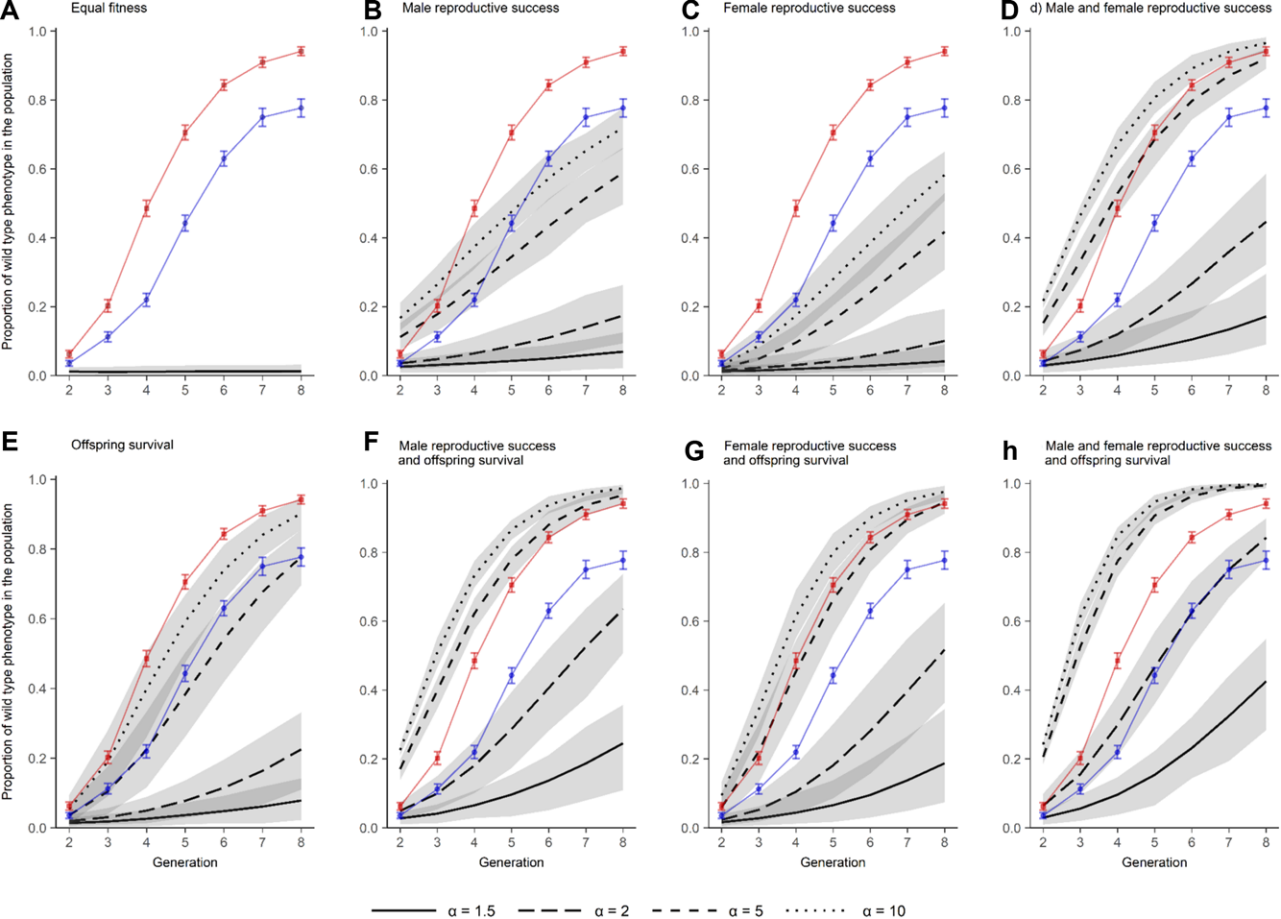
**Population genetic model predictions of the spread of the wild type (*WT*) phenotype into the intra‐specific competitor population of Reindeer (*Rd*) marker phenotype**. Model output compares the fitness advantage of the *WT* relative to the *Rd* genetic background (α) depending on contributions from adult male, adult female, and/or juvenile fitness. Lines and shaded areas represent medians and 5–95% quantiles, respectively, from 500 simulations. The simulations are overlaid with the experimental data for Monogamous (blue) and Polyandrous (red) sexual selection regimes.

Population genetic simulations confirmed that, with no fitness advantage of *WT* compared to *Rd* genotypes, little to no observations of the *WT* phenotype would be expected through our experiment (Fig. 4a). Thus, it was evident that the *WT* background, despite exhibiting 25% lower reproductive output in noncompetitive breeding conditions, had superior total fitness compared with the *Rd* population. Importantly, under modeling scenarios where the only fitness advantage of the *WT* genotype was via male reproductive success (Fig. 4b), we could not replicate the levels, rates, and extent of introgression observed in the experiment, even when assuming extreme differences in fitness (α, a 10‐fold fitness advantage). A similar scenario applied for explanations based solely on female reproductive success (Fig. 4c vs. 4b). When we simulated an advantage in male reproductive success alone, or male and female reproductive success combined, at the much higher values of α required to match the observed introgression data, we consistently found that higher proportions of *WT* phenotypes would then be expected in the early generations, conflicting with our empirical data (Fig. 4b and 4d).

The simulations that best matched the observed data were ones in which either there were higher values of α but only selection on offspring fitness (Fig. 4e), or intermediate values of α and selection on offspring fitness and female reproductive success (Fig. 4g), or where all three fitness drivers of male and female reproduction and offspring performance and viability were combined (Fig. 4h). Repeating our simulations allowing either 10 or 50% of offspring to survive to adulthood, representing lower and upper estimates of survival based on number of adults per gram of flour (Park 1948; Sokoloff 1972), yielded no qualitative difference in results (Fig. S1 and S2).

## Discussion

We find that the strength of sexual selection applied through experimental evolution influences a lineage's ability to invade into conspecific competitor populations. Although our sexually selected wild type *T. castaneum* strains showed overall superior fitness through the invasion challenge compared to their Reindeer mutant marker competitor, invasion was significantly faster and more complete following introductions from Polyandrous selection regime backgrounds (Fig. 2). Despite only comprising 10% of the population at initial colonization, we found that invading genomes from strong sexual selection backgrounds had almost completely introgressed and extirpated the Reindeer competitor genotypes by the eighth generation of the experiment, whereas invasions from weak Monogamous sexual selection histories plateaued at a level where they could not introgress above about three‐quarters of the population (Fig. 2). We used the dominant Reindeer marker to track introgression; *Rd* is a spontaneous and fully viable mutation in *T. castaneum* (Dawson 1984). Although successfully used as an informative paternity marker (e.g., Lewis et al. 2005; Tregenza et al. 2009; Michalczyk et al. 2011; Godwin et al. 2017), the requirement to maintain homozygous *Rd* strains can reduce general egg‐to‐adult fitness compared with heterozygotes (e.g., Lewis et al. 2005), which may explain the overall superiority of the sexually selected wild type strains through invasion (Fig. 2). As the wild type strains successfully introgressed into the Reindeer lineages (Fig. 2), recombination and gene flow through each generation will gradually decouple the *Rd* genetic marker from its original genetic background. Thus, by the end of the multi‐generation invasion experiment there is a reduced probability that the Reindeer (or wild type) phenotype and its state at the *Rd* locus is associated with its original genetic background. This decreasing linkage between the marker state and its original genetic background, however, applies equally to both Monogamous and Polyandrous background invaders. There is no evidence that the *WT* allele at the *Rd* locus behaves differently to the rest of the genome in a way that would bias the invasion rates by our different sexual selection backgrounds; if the *WT* allele could displace the *Rd* mutation at this one locus at a greater rate than introgression by the rest of the wild type genome, we would not expect the independent lines from the Monogamous wild type backgrounds to consistently reach an invasion plateau that is well below the near‐complete saturation shown by Polyandrous background invasions (Fig. 2).

Invasion ability provides a relevant test of a lineage's overall net fitness, because successful introgression through multiple generations will require populations to have combined superiority across all traits, in both sexes, and at all life stages. To understand which particular traits were more likely to explain the superiority of lineages from stronger sexual histories to colonize, establish, invade, and then introgress into conspecific competitor populations over multiple generations, we ran two sets of additional tests following the invasion assay. First, we checked for simple fertility and/or fecundity differences between the Monogamous and Polyandrous background adults, following mating and reproduction under benign conditions that applied no competition between adults or offspring. Given the known focus and impact of sexual selection on male mating and fertilising ability (e.g., Tilszer et al. 2006; Simmons and García‐González 2008; Nandy et al. 2013; Godwin et al. 2017), we wanted to check whether a selection history of enforced monogamy had not reduced the basic ability of males to mate and fertilize females, which could explain their weaker invasion ability. Results showed that Monogamous males possessed the same reproductive output in noncompetitive situations as Polyandrous regime males (Fig. 3), whether crossed with their own sexual selection background (Fig. 3b) or with a *Rd* female (Fig. 3c), so reduced invasion following weak sexual selection was not simply driven by reduced Monogamous male reproductive potential. In addition, we also checked for hybrid incompatibility or sterility between the wild type and Reindeer mutant crosses, and found no evidence, in the absence of competition, for any barriers to introgression in either crossing direction (Fig. 3d and 3e). Interestingly, within these noncompetitive fecundity assays, we also found that Reindeer mutant pairs could produce ∼25% more offspring than wild type adults from either Monogamous or Polyandrous backgrounds (Fig. 3a). This superior fecundity in the absence of competition between adults or juveniles clearly did not translate into wider fitness within the more ecologically and evolutionarily relevant demands of the invasion challenge, where the Reindeer mutant genotypes showed reduced fitness compared to all wild type genetic backgrounds when competing to survive, reproduce, and introgress.

Having established that there was equivalent reproductive potential within adults of both sexual selection backgrounds, and that there were no hybridization barriers to introgression between wild type (*WT*) and Reindeer (*Rd*) strains, we ran population genetic simulations to distinguish between the relative contributions of (1) adult male reproductive output, (2) adult female reproductive output, and (3) offspring fitness (and combinations of all three possible drivers). Knowledge of the genetic basis of the *Rd* homozygous dominant mutant marker (Dawson 1984), the composition of the populations at the start of the experiment, the design of the invasion experiment, and the shapes of the different sexual selection regime introgression curves fitted to the empirical data (Fig. 2), allowed simulations to reveal the most probable explanations for our results. Specifically, we simulated how invasion rate would be affected by overall fitness differences between the *WT* and *Rd* genotypes, and then how these fitness differentials impacted on invasion rates if they were the specific consequence of (1) relative reproductive success by *WT* males, (2) relative reproductive success by *WT* females, or (3) relative offspring performance and survival to adulthood by *WT* juveniles (or different combinations of these three drivers; Fig. 4). Because we began the invasion experiment by introducing only male *WT* invaders, any contribution to invasion from variation in female *WT* reproductive success or offspring performance will lag behind male effects, allowing the simulations to differentiate between impacts of sex‐specific reproduction and overall offspring viability, especially in the early stages of the experiment. Applying this logic, and simulating the structure and design of our experiment (see Methods), the simulation results indicated that differences in invasion rates between weak and strong sexual selection *WT* backgrounds cannot be correlated with variance in male reproductive success alone (Fig. 4b). Indeed, neither the relative success of either adult male or adult female reproductive success is consistent with our invasion and introgression findings, unless we also incorporate variance in offspring performance and viability into our simulations. If we assume that the fitness differential between *WT* and *Rd* genotypes (α) is very large within the competitive invasion challenge, and there is least a 10‐fold difference in overall fitness between *WT* and *Rd* juveniles, then an explanation based upon variation in offspring fitness and survival alone is most consistent with our invasion results, without any differential contribution from adult reproduction at all (see matches between the empirical data and simulation where α = 10 in Fig. 4e). However, if we assume a lower, twofold α fitness differential between *WT* and *Rd* genotypes (which may be reasonable given the higher fecundity shown by *Rd* pairs, albeit within benign, noncompetitive conditions, Fig. 3a), a simulation that is driven by fitness variance in all three drivers of (1) male and (2) female reproductive success, and (3) overall offspring performance and viability, is most consistent with our invasion assay results (see matches between the empirical data and simulation where α = 2 in Fig. 4h). Whichever overall fitness differential between *WT* and *Rd* applies in reality, these models indicate that the empirical invasion results are most consistent with our simulations where there is improved fitness across all life stages in both sexes, and not just adult male reproduction.

Our combined results support genic capture theory (Rowe and Houle 1996) proposing that sexual selection acts on a broad range of traits that improve the ability of individuals to maintain condition, and not just competitive success in male courtship, mating, and fertilization (Rowe and Houle 1996; Lorch et al. 2003; Tomkins et al. 2004; Whitlock and Agrawal 2009). Although male mating or sperm competition success are known to respond to experimental evolution under sexual selection (Tilszer et al. 2006; Simmons and García‐González 2008; Nandy et al. 2013), including in *T. castaneum* (Michalczyk et al. 2011; Demont et al. 2014; Godwin et al. 2017), our simulations and experimental design suggest that these male traits alone could not explain the superior invasion ability of our Polyandrous regime populations. Whatever fitness differential between *WT* and *Rd* males we apply within competitive reproduction, the simulation cannot fit the empirical data (see Fig. 4b and Fig. S1b); if *WT* males have a 1.5‐ or twofold advantage in reproductive competition over *Rd* males, predicted invasion rates proceed much below the empirical results. If the fitness differential between *WT* and *Rd* males is large (where α assumes a 5‐ or 10‐fold advantage of *WT* over *Rd* males), then simulated invasion rates by the end of the experiment are closer to the empirical data but, importantly, invasion levels of *WT* invaders at the start of the experiment are predicted to be much higher than our empirical results show.

Previous empirical research using different approaches to measure the population‐level fitness consequences of sexual selection has yielded mixed findings. Sexual selection was found to improve some important fitness traits and reduce the build‐up of mutation load (Jarzebowska and Radwan 2010; McGuigan et al. 2011), as well as effectively removing experimentally introduced mutations (Radwan 2004; Hollis et al. 2009; Almbro and Simmons 2014) having sex‐specific effects on male reproductive fitness (Grieshop et al. 2016). However, similar studies have found either neutral (Arbuthnott and Rundle 2012; Power and Holman 2015) or negative effects (Hollis and Houle 2011) on the recovery of fitness through sexual selection. In a mutation accumulation study with *D. serrata*, sexual selection did not prevent fitness reductions associated with the buildup of load, but did strengthen the fitness correlation between males and females, indicating an importance for sex‐limited genetic load for population fitness (Allen et al. 2017). Evidence that sexual selection helps to fix beneficial alleles more effectively when environments change also varies, with some studies reporting that sexual selection improves adaptation to a novel environment (Fricke and Arnqvist 2007), aids the evolution of pesticide resistance (Jacomb et al. 2016), or prevents extinction (Plesnar‐Bielak et al. 2012). However, others have found no equivalent benefit (Holland 2002; Rundle et al. 2006) or even antagonism against adaptation (Chenoweth et al. 2015). Some of these inconsistencies may be explained by the measurement of effects on fitness in tandem with the action of sexual selection, where benefits could be confounded by the simultaneously negative shorter‐term action of sexual conflict (Parker 1979; Whitlock and Agrawal 2009; Holman and Kokko 2013). To overcome this possible confound, Lumley et al. (2015) examined evidence for relative differences in genetic load in the *Tribolium castaneum* populations used in the current study using an inbreeding assay: having removed any simultaneous actions of sexual conflict, results showed that sexual selection improved resistance to inbreeding depression and extinction risk. In this new study, we employ an invasion assay that tests for net fitness variance in phenotypes across all life stages in both sexes and through multiple generations; in combination with population genetic simulations, we find broad and positive influences of sexual selection for both sexes and juvenile life stages.

Successful invasion over multiple generations in the face of conspecific competition will require superior fitness across a range of traits, in both sexes, and through all life stages. The invasive superiority shown by our Polyandrous background populations could have evolved if sexual selection acts as an effective intraspecific filter for purging mutation load and/or fixing beneficial alleles widely across the genome within lineages (Rowe and Houle 1996; Manning 1984; Whitlock 2000; Agrawal 2001; Siller 2001; Lorch et al. 2003; Whitlock and Agrawal 2009). By contrast, the removal of sexual selection across 77 generations of enforced monogamy may have relaxed selection on beneficial alleles and/or allowed mutation load to accumulate across the genome, reducing the subsequent ability of these lineages to invade and introgress against conspecific competitor genotypes. Mutation accumulation experiments over similar timescales to our own period of experimental evolution show that the buildup of fitness‐reducing genetic load can occur under relaxed selection, even in smaller populations across tens of generations (Mukai 1964; Halligan and Keightley 2009; McGuigan et al. 2011; Charlesworth 2015), including those of *T. castaneum* (Enfield and Braskerud 1989).

Although invasion success will depend upon a number of abiotic and biotic drivers, an important determinant of invasiveness is when more efficient and successful invaders decrease resource availability for those competitors less proficient at gaining it, accentuating fitness differences between groups (Begon et al. 2009). Modeling shows that even small differences in mutation load between closely‐related or ecologically analogous species can easily lead to extinction through competitive exclusion of the species carrying slightly higher load (Agrawal and Whitlock 2012). Competitor and ‘hybrid’ genotypes that are most efficient at acquiring, managing, and using resources, are therefore predicted to dominate (Tilman 1982; Shea and Chesson 2002). *T. castaneum* ecology and life history is strongly influenced by density dependent competition for food and growth through the juvenile stages, while avoiding disease and cannibalism, followed by eventual competition for reproduction among adults (Sokoloff 1972). Both larvae and adults live within their food (flour, yeast, and oats), but need to move through the fodder to locate areas that have not been already used and spoiled (Sokoloff 1972). Optimal foraging will improve development, and the avoidance of degraded food resources potentially containing pathogens (Via 1999). Faster development at the front of the cohort will also avoid cannibalism by more advanced life stages (Sokoloff 1972). Results from our empirical and simulation approaches indicate that sexual selection has influenced these nonreproductive traits, despite them being traditionally viewed as the focus of natural selection.

## DATA ACCESSIBILITY

Data available from the Dryad Digital Repository (data identifiers TBC).

## CONFLICT OF INTEREST

The authors declare no conflict of interest.

Associate Editor: R. Snook

## Supporting information


**Figure S1**. Population genetic model predictions of the spread of the wild type (*WT*) phenotype into the intra‐specific competitor population of Reindeer (*Rd*) marker phenotype with 10% offspring survival to adulthood.
**Figure S2**. Population genetic model predictions 10 of the spread of the wild type (*WT*) phenotype into the intra‐specific competitor population of Reindeer (*Rd*) marker phenotype with 50% offspring survival to adulthood.
