# Supplementary material for: Lineages evolved under stronger sexual selection show superior ability to invade conspecific competitor populations
Source: Evol Lett. 2018 Aug 16;2(5):511–23. doi: 10.1002/evl3.80 (PMC6145403; doi:10.1002/evl3.80)
Supplement: Supplementary file 1 — Figure S1. Population genetic model predictions of the spread of the wild type (WT) phenotype into the intra‐specific competitor population of Reindeer (Rd) marker phenotype with 10% offspring survival to adulthood. Figure S2. Population genetic model predictions 10 of the spread of the wild type (WT) phenotype into the intra‐specific competitor population of Reindeer (Rd) marker phenotype with 50% offspring survival to adulthood. [file EVL3-2-511-s001.pdf]

Supporting Information

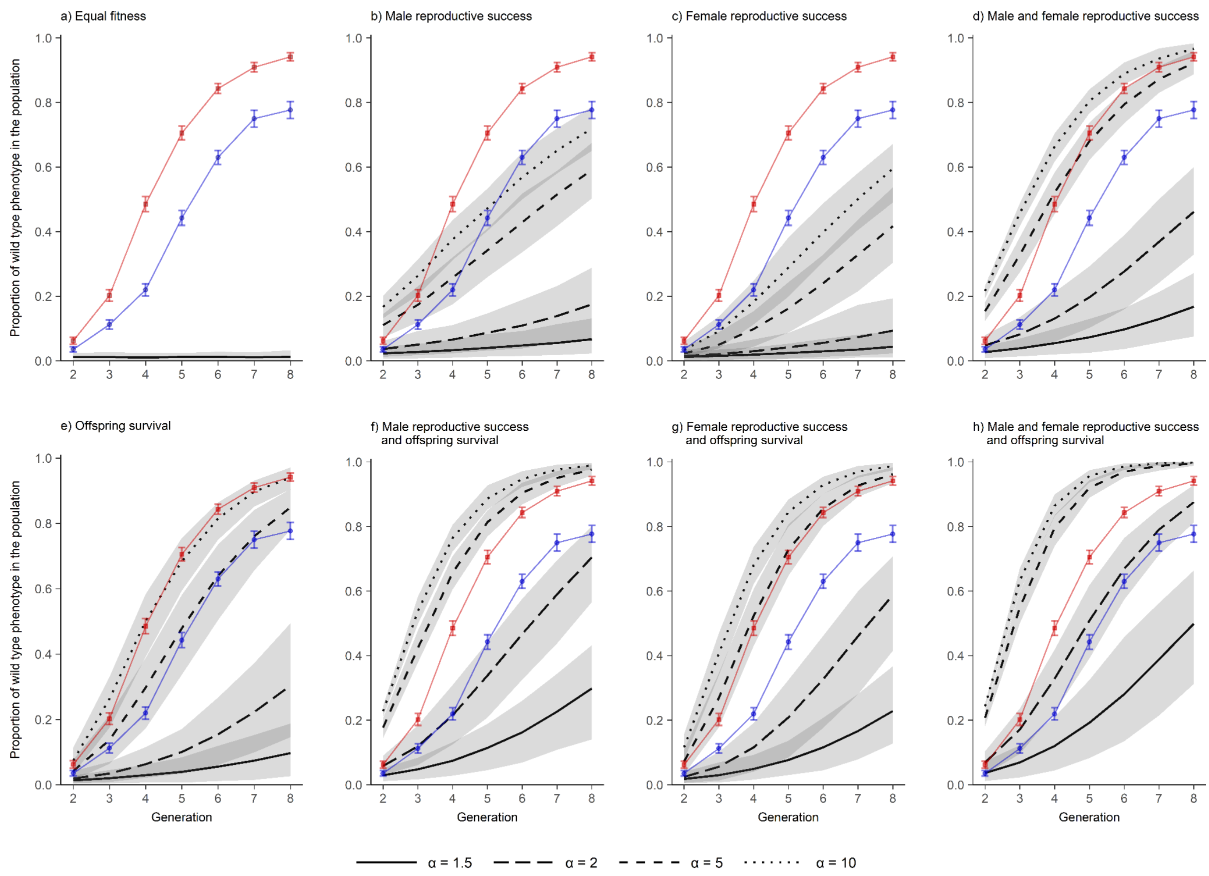

Supporting Information Figure S1 | Population genetic model predictions of the spread of the wild type (*WT*) phenotype into the intra-specific competitor population of Reindeer (*Rd*) marker phenotype with 10% offspring survival to adulthood. Model output compares the fitness advantage of the *WT* relative to the *Rd* genetic background (α) depending on contributions from adult male, adult female and/or juvenile fitness. Lines and shaded areas represent medians and 5%-95% quantiles, respectively, from 500 simulations. The simulations are overlaid with the experimental data for Monogamous (blue) and Polyandrous (red) sexual selection regimes.

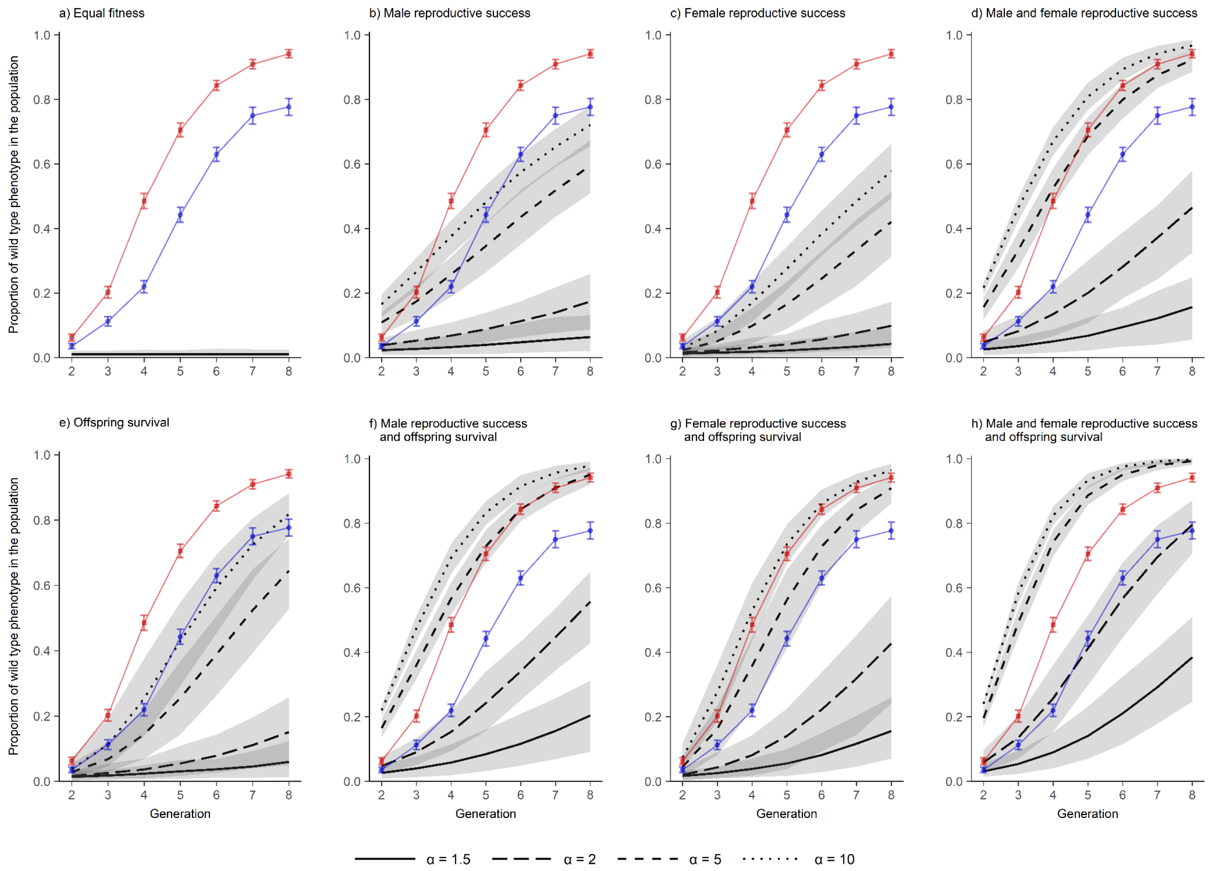

Supporting Information Figure S2 | Population genetic model predictions of the spread of the wild type (*WT*) phenotype into the intra-specific competitor population of Reindeer (*Rd*) marker phenotype with 50% offspring survival to adulthood. Model output compares the fitness advantage of *WT* relative to the *Rd* genetic background (α) depending on contributions from adult male, adult female and/or juvenile fitness. Lines and shaded areas represent medians and 5%-95% quantiles, respectively, from 500 simulations. The simulations are overlaid with the experimental data for Monogamous (blue) and Polyandrous (red) sexual selection regimes.
